# Identification of Tumor Microenvironment-Related Prognostic Biomarkers in Luminal Breast Cancer

**DOI:** 10.3389/fgene.2020.555865

**Published:** 2020-11-10

**Authors:** Yanyan Wang, Mingzhi Zhu, Feng Guo, Yi Song, Xunjie Fan, Guijun Qin

**Affiliations:** ^1^Department of Breast Surgery, The First Affiliated Hospital of Zhengzhou University, Zhengzhou, China; ^2^Department of Endocrinology, The First Affiliated Hospital of Zhengzhou University, Zhengzhou, China

**Keywords:** luminal breast cancer, tumor microenvironment, ESTIMATE algorithm, messenger RNA, microRNA, long noncoding RNA

## Abstract

**Background:** The tumor microenvironment (TME) has been reported to have significant value in the diagnosis and prognosis of cancers. This study aimed to identify key biomarkers in the TME of luminal breast cancer (BC).

**Methods:** We obtained immune scores (ISs) and stromal scores (SSs) for The Cancer Genome Atlas (TCGA) luminal BC cohort from the online ESTIMATE (Estimation of STromal and Immune cells in MAlignant Tumor tissues using Expression data) portal. The relationships between ISs and SSs and the overall survival of luminal BC patients were assessed by the Kaplan-Meier method. The differentially expressed messenger RNAs (DEmRNAs) related to the ISs and SSs were subjected to functional enrichment analysis. Additionally, a competing endogenous RNA (ceRNA) network was constructed with differentially expressed microRNAs (DEmiRNAs) and long noncoding RNAs (DElncRNAs). Furthermore, a protein–protein interaction (PPI) network was established to analyze the DEmRNAs in the ceRNA network. Then, survival analysis of biomarkers involved in the ceRNA network was carried out to explore their prognostic value. Finally, these biomarkers were validated using the luminal BC dataset from the Gene Expression Omnibus (GEO) database.

**Results:** The results showed that ISs were significantly associated with longer survival times of luminal BC patients. Functional enrichment analysis showed that the DEmRNAs were mainly associated with immune response, antigen binding, and the extracellular region. In the PPI network, the top 10 DEmRNAs were identified as hub genes that affected the TME of luminal BC. Finally, two DEmiRNAs, two DElncRNAs, and 17 DEmRNAs of the ceRNA network associated with the TME were shown to have prognostic value. Subsequently, the expression of 15 prognostic biomarkers was validated in one additional dataset (GSE81002). In particular, one lncRNA (GVINP1) and five mRNAs (CCDC69, DOCK2, IKZF1, JCHAIN, and NCKAP1L) were novel biomarkers.

**Conclusions:** Our studies demonstrated that ISs were associated with the survival of luminal BC patients, and a set of novel biomarkers that might play a prognostic role in the TME of luminal BC was identified.

## Introduction

Breast cancer (BC) is one of the most common malignant tumors and the main cause of cancer-associated death in women worldwide. Treatment measures for BC patients, such as surgical methods and drug regimens, have been constantly improved, while the clinical outcomes of individual patients remain difficult to predict (Clough et al., 2010; Graham et al., 2015). BC is a heterogeneous disease in terms of cellular composition, molecular alterations, and clinical outcomes within different tumor subtypes and within a single tumor. BC is commonly categorized by gene expression profiling into four main subtypes: luminal A, luminal B, human epidermal growth factor receptor 2 (HER2)-enriched, and triple-negative (TN; Lorona et al., 2019). More than 70% of the diagnosed BC cases are the luminal subtype and positive for estrogen receptor and/or progesterone receptor (ER+ and PR+, respectively; Polyak and Metzger Filho, 2012). Although the recurrent risk of luminal BC patients can be reduced by endocrine therapy, some patients still relapse after 5 years.

In recent years, an increasing number of studies have focused on the tumor microenvironment, which consists of immune cells, stromal cells, endothelial cells, mesenchymal cells, inflammatory mediators, extracellular matrix molecules, and numerous cytokines and chemokines (Hanahan and Coussens, 2012; De Nola and Menga, 2019). Among them, immune cells and stromal cells function as two essential components of the tumor microenvironment (TME) and have been reported to have significant value in the diagnosis and prognosis of cancers. ESTIMATE (Estimation of Stromal and Immune cells in Malignant Tumor tissues using Expression data) is an algorithm that can be used to predict the infiltration of stromal and immune cells and their prognostic value by accessing the stromal scores (SSs) and immune scores (ISs) in tumors based on RNA sequencing (RNA-Seq) data extracted through The Cancer Genome Atlas (TCGA) database (Yoshihara et al., 2013). To date, several studies have investigated the microenvironment of many cancers, including BC and TNBC (Deng and Lu, 2019; Wang et al., 2019). For luminal BC, Li and Sang (2020) mainly investigated DNA methylation and key genes that regulate immune cell infiltration in luminal BC. Furthermore, the roles of competing endogenous RNAs (ceRNAs) [long noncoding RNAs (lncRNAs) sharing microRNA (miRNA) response elements (MREs) with messenger RNAs (mRNAs)] associated with the ISs and/or SSs of luminal BC have not been investigated.

LncRNAs are defined as RNA transcripts of more than 200 nucleotides and were once considered transcriptional “noise” without protein-coding capacity (Jathar et al., 2017). They play a fundamental role in the interaction between cancer cells and the surrounding environment during tumor progression (Sun et al., 2018). Recent studies have suggested that lncRNAs, as regulators, play important roles in cancer immune processes, such as infiltration into cancer tissues, immune activation, and immune cell migration (Carpenter and Fitzgerald, 2018; Denaro et al., 2019). For example, hepatoma cell lines overexpressing the lncRNA HOTAIR secreted more C-C motif chemokine ligand 2 (CCL2) than control cell lines, which promoted the proliferation of tumor-associated macrophages and myeloid-derived suppressor cells (Botti et al., 2019). In addition, studies have also shown that an immune-related lncRNA signature has important value for survival prediction in several cancers, including diffuse large B cell lymphoma, hepatocellular carcinoma, non-small-cell lung cancer (NSCLC), and BC (Zhou et al., 2017; Shen et al., 2020; Sun et al., 2020; Zhang et al., 2020).

In the current study, by taking advantage of the RNA-Seq and clinical data of 566 luminal BC tumor samples from TCGA database and the ESTIMATE algorithm, we aimed to explore the association between overall survival and ISs and SSs of luminal BC and construct a ceRNA network of differentially expressed mRNAs (DEmRNAs), lncRNAs (DElncRNAs), and miRNAs (DEmiRNAs) related to the ISs and SSs. Then, we compiled a list of TME-related biomarkers that were associated with the survival of luminal BC patients. Finally, these biomarkers were validated using the luminal BC dataset from the Gene Expression Omnibus (GEO) database. Thus, our study might help better understand the molecular mechanisms in the TME of luminal BC and identify potential immune biomarkers that predict the survival outcomes of patients.

## Materials and Methods

### Data Processing and Estimation of Immune Scores and Stromal Scores

In order to provide an overall estimation of the molecular mechanisms in the TME of luminal BC, we combined RNA-Seq datasets and clinical information of luminal A and luminal B patients from TCGA database.^1^ The inclusion criteria were as follows: (1) patients who had no other tumors, (2) samples with mRNA, miRNA, and lncRNA sequencing data, and (3) luminal A and luminal B subtypes classified by PAM50 gene expression profile. Finally, 556 luminal BC patients were finally included in this study. The ESTIMATE algorithm was used to estimate the ISs and SSs of each luminal BC sample. The gene expression profiles of the GSE81002 cohort were downloaded from the GEO database for validation.^2^

### Association Between Immune Scores/Stromal Scores and the Prognoses of Luminal Breast Cancer Patients

According to each sample IS and SS, all luminal BC patients were classified into high‐ and low-score groups. The overall survival of these two groups was estimated with the Kaplan-Meier survival estimator, and the corresponding survival outcomes of the two groups were compared by log-rank tests.

### Identification of Differentially Expressed mRNAs, miRNAs, and lncRNAs Based on Immune Scores and Stromal Scores

By comparing the two groups above, DEmRNAs, DEmiRNAs, and DElncRNAs were filtered with the cutoff criteria of *p* < 0.05, false discovery rate (FDR) <0.05, and fold change (FC) >1.2 by using the R package limma. Venn diagrams were generated to show the intersection between the DEmRNAs, DEmiRNAs, and DElncRNAs of both the IS and SS groups.

### Functional Enrichment Analysis

We used the Database for Annotation, Visualization, and Integrated Discovery (DAVID) online tool to perform Gene Ontology (GO) enrichment and Kyoto Encyclopedia of Genes and Genomes (KEGG) analyses to explore the functional roles of intersecting DEmRNAs (Ashburner et al., 2000; Kanehisa, 2002).^3^ The GO terms are grouped by biological processes (BPs), molecular functions (MFs), and cellular components (CCs). *p* < 0.05 was considered statistically significant.

### Construction of the Competing Endogenous RNA and Protein: Protein Interaction Networks

The target genes and lncRNAs of the overlapping DEmiRNAs were predicted. The target genes were predicted using the MiRanda and TargetScan algorithms, and the target lncRNAs were predicted through the MiRanda and PITA algorithms. Out of all the predictions, only the target mRNAs and lncRNAs predicted in both algorithms were considered candidates, and the common mRNAs and lncRNAs with the IS/SS-associated overlapping DEmRNAs and DElncRNAs mentioned above were chosen to construct the ceRNA network.

The DEmRNAs involved in the ceRNA network mentioned above were selected to construct the protein–protein interaction (PPI) network by using the Search Tool for the Retrieval of Interacting Genes (STRING) database.^4^ The resulting network was visualized with Cytoscape software (version 3.6.1).

### Prognostic Value of Biomarkers Involved in the Competing Endogenous RNA Network

Based on their median survival time, luminal BC patients were divided into high-risk and low-risk groups. Kaplan-Meier survival analysis and the log-rank test were used to assess the relationship between patient overall survival and the expression levels of DEmiRNAs, DEmRNAs, and DElncRNAs that were involved in the ceRNA network. *p* < 0.05 was considered statistically significant.

### Statistical Analysis

R version 3.5.0 was used to conduct statistical analyses. The figures were generated using related packages including limma, heatmap, survival, and so on. *p* < 0.05 was considered statistically significant.

## Results

### The Prognostic Value of Immune Scores and Stromal Scores in Luminal Breast Cancer Patients

The RNA sequencing datasets downloaded from TCGA database consisted of 556 luminal BC samples, of which 381 were luminal A and 175 were luminal B samples. By using the ESTIMATE algorithm, we found that the ISs of the patients ranged from −1,276.49 to 3,801.82, and the SSs ranged from −2,204.39 to 2,216.92.

**Figure 1:**
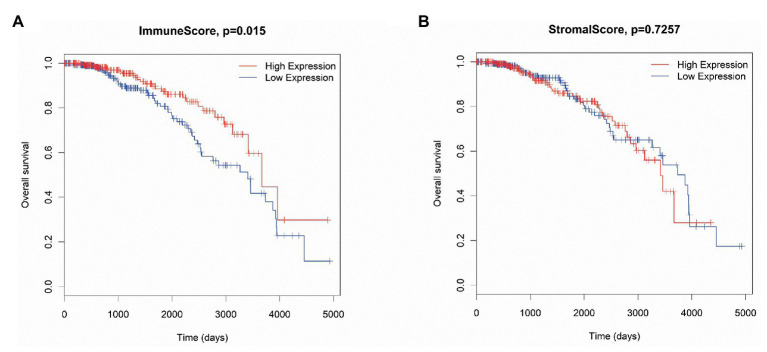
The association between immune scores (ISs; **A**) and stromal scores (SSs; **B**) and overall survival in luminal breast cancer (BC) patients.

Additionally, to determine the association between ISs and SSs and the overall survival time of luminal BC patients, the patients were divided into high‐ and low-score groups. Then, the patient data were analyzed by Kaplan-Meier survival analysis. The results indicated that patients with high ISs had longer survival times than patients with low ISs (*p* = 0.015; Figure 1A). However, there was no difference between the high-SS group and the low-SS group (Figure 1B).

### Identification of Differentially Expressed mRNAs, miRNAs, and lncRNAs Based on Immune Scores and Stromal Scores

**Figure 2:**
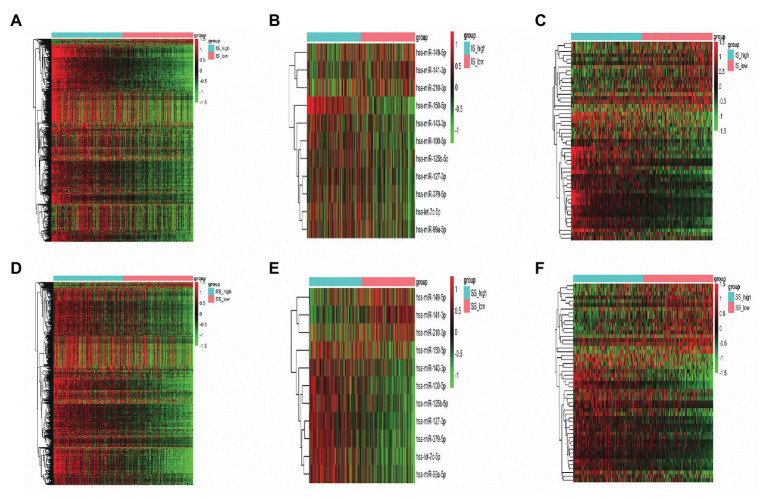
**(A–C)** Heatmaps of differentially expressed messenger RNAs (DEmRNAs), microRNAs (DEmiRNAs), and long noncoding RNAs (DElncRNAs) in the high‐ vs. low-immune score (IS) groups. **(D–F)** Heatmaps of DEmRNAs, DEmiRNAs, and DElncRNAs in the high‐ vs. low-stromal score (SS) groups. Green represents high expression, and red represents low expression.

**Figure 3:**
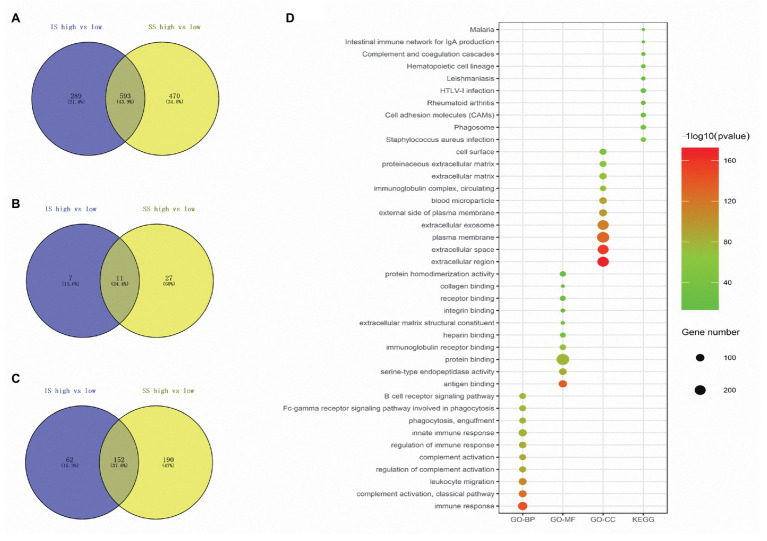
**(A–C)** Venn diagrams showing the number of commonly regulated differentially expressed messenger RNAs (DEmRNAs), microRNAs (DEmiRNAs), and long noncoding RNAs (DElncRNAs) in the immune score (IS) and stromal score (SS) groups. **(D)** Biological process (BP), molecular function (MF), cellular component (CC), and pathway terms of the top 10 upregulated DEmRNAs.

In total, 882 and 1,063 DEmRNAs, 18 and 38 DEmiRNAs, and 214 and 342 DElncRNAs were identified based on the comparison of the high‐ vs. low-IS and high‐ vs. low-SS groups, respectively (Figures 2A–F). Moreover, through Venn diagram constructions, 593 common DEmRNAs (577 upregulated and 16 downregulated), 11 common DEmiRNAs (eight upregulated and three downregulated), and 152 common DElncRNAs (93 upregulated and 59 downregulated) were obtained (Figures 3A–C). Subsequently, these common DEmRNAs, DEmiRNAs, and DElncRNAs were used for further analysis.

### Functional Enrichment Analysis

Among the common DEmRNAs, only 16 DEmRNAs were downregulated; thus, we only chose 577 co-upregulated DEmRNAs for biological function exploration, and the data are presented in three subontologies: BP, CC, and MF. With regard to the BP category, the upregulated DEmRNAs were mainly enriched in immune response, complement activation, and leukocyte migration. For the MF category, the upregulated DEmRNAs were mainly involved in antigen binding, serine-type endopeptidase activity, and protein binding. The upregulated DEmRNAs were mainly associated with the extracellular region, extracellular space, and plasma membrane terms in the CC category. In addition, according to the pathway analysis, the upregulated DEmRNAs were mainly associated with *Staphylococcus aureus* infection, phagosomes, and cell adhesion molecules (CAMs). The top 10 GO and KEGG pathway terms of the co-upregulated DEmRNAs are shown in Figure 3D.

### Construction of the Competing Endogenous RNA and Protein: Protein Interaction Networks

**Figure 4:**
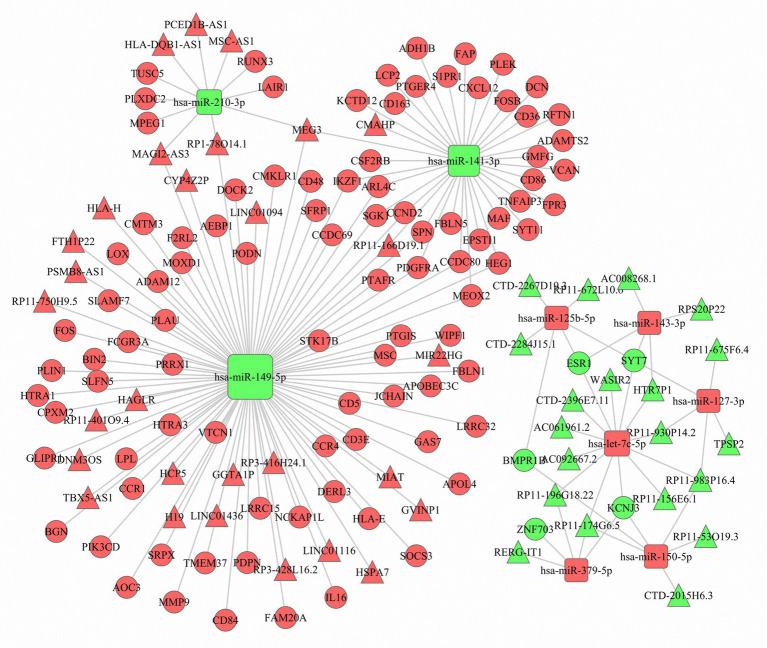
The competing endogenous RNA (ceRNA) network. Red represents upregulation, and green represents downregulation. The circle nodes represent differentially expressed messenger RNAs (DEmRNAs), the rectangle nodes denote differentially expressed microRNAs (DEmiRNAs), and the triangle nodes represent differentially expressed long noncoding RNAs (DElncRNAs).

Based on the negatively regulated miRNA-mRNA and miRNA-lncRNA pairs, a ceRNA network was constructed with 99 DEmRNAs, nine DEmiRNAs, and 49 DElncRNAs (Figure 4). The nine DEmiRNAs (hsa-miR-149-5p, hsa-miR-141-3p, hsa-let-7c-5p, hsa-miR-210-3p, hsa-miR-125b-5p, hsa-miR-150-5p, hsa-miR-127-3p, hsa-miR-143-3p, and hsa-miR-379-5p) were highly correlated with other DEmRNAs and DElncRNAs. For instance, hsa-miR-149-5p, which acted as a ceRNA, was correlated with 65 DEmRNAs and 25 DElncRNAs. This means that the expression of these hub miRNAs might be regulated by a number of lncRNAs and mRNAs to affect the TME of luminal BC.

**Figure 5:**
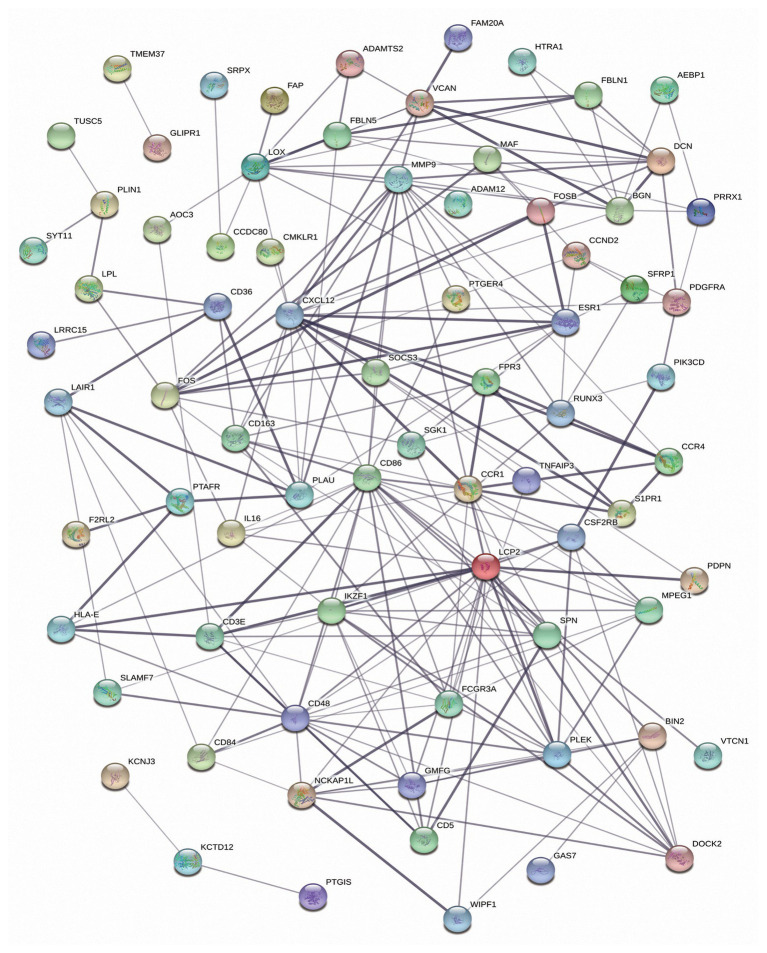
The protein-protein interaction (PPI) network of the differentially expressed mRNAs (DEmRNAs) in the competing endogenous RNA (ceRNA) network.

Next, to better understand the interactions among DEmRNAs of the ceRNA network, a PPI network was constructed with 75 nodes and 215 edges. The top 10 DEmRNAs (CD86, LCP2, CXCL12, MMP9, CD48, CCR1, IKZF1, PLEK, LOX, and ESR1) with the highest degree of connectivity were identified as hub genes in the PPI network (Figure 5).

### Prognostic Values of Biomarkers Involved in the Competing Endogenous RNA Network

**Figure 6:**
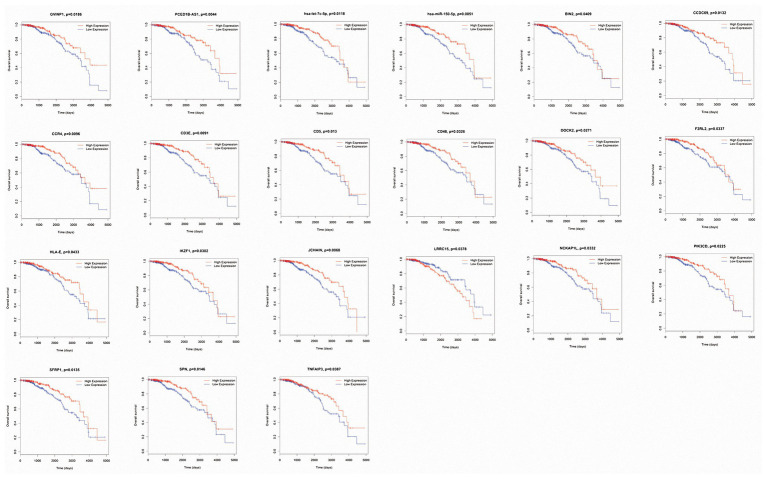
Kaplan-Meier survival curves of the differentially expressed microRNAs (DEmiRNAs), long noncoding RNAs (DElncRNAs), and messenger RNAs (DEmRNAs) significantly associated with overall survival in luminal breast cancer (BC) patients.

Ninety-nine DEmRNAs, nine DEmiRNAs, and 49 DElncRNAs in the ceRNA network were included in the survival analysis. Two DElncRNAs (GVINP1 and PCED1B-AS1), two DEmiRNAs (hsa-let-7c-5p and hsa-miR-150-5p), and 17 DEmRNAs (BIN2, CCDC69, CCR4, CD3E, CD5, CD48, DOCK2, F2RL2, HLA-E, IKZF1, JCHAIN, LRRC15, NCKAP1L, PIK3CD, SFRP1, SPN, and TNFAIP3) were found to be closely related to the overall survival of luminal BC patients (*p* < 0.05; Figure 6). Of these survival-associated biomarkers, only the high expression of LRRC15 was related to unfavorable survival outcomes of luminal BC patients. For the rest of the biomarkers, high expression levels were associated with favorable survival outcomes.

### Validation in the Gene Expression Omnibus Database

To determine whether these survival-associated biomarkers were also of prognostic significance in an independent database, a cohort of 247 luminal BC cases was downloaded from the GEO database (GSE81002). However, due to the lack of follow-up information, we only validated their differential expression levels between the high‐ and low-IS/SS groups. The results showed that the expression levels of the lncRNA GVINP1 and 14 mRNAs (BIN2, CCDC69, CCR4, CD5, CD48, DOCK2, HLA-E, IKZF1, JCHAIN, LRRC15, NCKAP1L, PIK3CD, SPN, and TNFAIP3) were consistent in the two comparisons (all *p* < 0.05), which was consistent with the results for TCGA cohort (Supplementary Figures 1, 2). In particular, the lncRNA GVINP1 and five mRNAs (CCDC69, DOCK2, IKZF1, JCHAIN, and NCKAP1L) were identified as novel biomarkers.

## Discussion

An increasing amount of evidence has elucidated the clinicopathological significance of the TME in the prediction of treatment effects (Binnewies et al., 2018; Zeng et al., 2019). Evaluating the proportions of microenvironment-associated cell types may help explore the role of TME and provide perspectives in cancer research. Recently, researchers have suggested the clinical importance of stromal cells and immune cells in the microenvironment of BC tissues (Wang et al., 2019; Li and Sang, 2020). However, the roles of ceRNAs associated with the ISs and/or SSs of luminal BC have not been investigated. The ceRNA hypothesis suggests a novel regulatory mechanism that can be mediated by lncRNAs, and lncRNAs with sequences similar to those of their target miRNAs are able to regulate the expression of mRNAs by acting as sponges for miRNAs (Salmena et al., 2011).

In this study, we evaluated the ISs and SSs of luminal BC patients. Our results showed that high ISs were significantly associated with longer survival time of patients than low ISs, which was consistent with a previous study that reported on IS evaluation in BC patients (Wang et al., 2019). This might be because higher ISs suggest enhanced immune system and function, which can be stimulated to increase the antitumor immunity of the TME to then control and eliminate the tumor (Blattman and Greenberg, 2004; Chen and Mellman, 2017). Furthermore, although no correlation between SSs and luminal BC patient survival was found, this did not necessarily mean that stroma-specific biomarkers are not associated with prognosis. Thus, we used the intersecting DEmRNAs, DEmiRNAs, and DElncRNAs obtained from the two groups (high vs. low ISs and high vs. low SSs) to explore their prognostic value in luminal BC patients.

A total of 593 DEmRNAs, 11 DEmiRNAs, and 152 DElncRNAs common between the three comparisons were selected for further analysis. Of the 593 shared DEmRNAs, 577 were upregulated, accounting for 97.3%; thus, we mainly focused on their biological functions. The results indicated that many of them were associated with TME-related processes, such as the immune response, leukocyte migration, antigen binding, and the CAM pathway (Harjunpaa et al., 2019; Cao et al., 2020; Messex et al., 2020; Sulea et al., 2020). Next, ceRNA and PPI networks were constructed. In the ceRNA network, nine DEmiRNAs (hsa-miR-149-5p, hsa-miR-141-3p, hsa-let-7c-5p, hsa-miR-210-3p, hsa-miR-125b-5p, hsa-miR-150-5p, hsa-miR-127-3p, hsa-miR-143-3p, and hsa-miR-379-5p) acted as ceRNAs that had important roles in regulating the network along with many other DEmRNAs and DElncRNAs. Among these miRNAs, hsa-let-7c-5p and hsa-miR-150-5p were associated with increased survival times of luminal BC patients. Additionally, 10 DEmRNAs (CD86, LCP2, CXCL12, MMP9, CD48, CCR1, IKZF1, PLEK, LOX, and ESR1) with the highest degree of connectivity were identified as hub genes in the PPI network. Only CD48 and IKZF1 were related to favorable survival outcomes. Apart from the abovementioned prognostic biomarkers, there were another two DElncRNAs (GVINP1 and PCED1B-AS1) and 15 DEmRNAs (BIN2, CCDC69, CCR4, CD3E, CD5, DOCK2, F2RL2, HLA-E, JCHAIN, LRRC15, NCKAP1L, PIK3CD, SFRP1, SPN, and TNFAIP3) that contributed to the overall survival of patients with luminal BC. Finally, we downloaded data from a luminal BC cohort to verify these biomarkers. We only successfully confirmed the differential expression of some of the RNAs and failed to detect whether they were associated with prognosis due to a lack of survival information. Of the 15 validated biomarkers, the lncRNA GVINP1 and five mRNAs (CCDC69, DOCK2, IKZF1, JCHAIN, and NCKAP1L) had not been previously reported in BC, but these independent factors might serve as prognostic biomarkers.

The role of lncRNAs in BC has been widely studied. The lncRNAs HIF1A-AS2 and AK124454 have been shown to promote cell proliferation and invasion in TNBC cells and contribute to paclitaxel resistance (Jiang et al., 2016). Four lncRNAs (ADAMTS9-AS1, LINC00536, AL391421.1, and LINC00491) have been shown to have a significant prognostic value, and an lncRNA signature containing these four lncRNAs independently predicted overall survival in BC patients (Fan et al., 2018). LncRNAs might also act as novel candidate biomarkers to identify BC patients at high risk of tumor recurrence (Zhou et al., 2016). In addition, Shen et al. (2020) observed that an 11-lncRNA prognostic signature for BC was associated with the infiltration of immune cell subtypes. In our study, only one lncRNA, GVINP1, was found to be associated with the overall survival in luminal BC. The lncRNA GVINP1 can bind with guanosine triphosphate selectively and noncovalently, and it has been found to be an independent prognostic marker for lung adenocarcinoma (LUAD) and NSCLC patients (Sui and Yang, 2019; Zhou et al., 2019). Our study also indicated that high expression of GVINP1 could compete with the downregulated miRNA hsa-miR-149-5p to regulate the expression of target genes, including several novel survival-associated genes such as CCDC69, DOCK2, IKZF1, JCHAIN, and NCKAP1L involved in the ceRNA network.

CCDC69 can act as a regulator of the formation of mitotic spindles and DNA replication in eukaryotic cells (Fu et al., 2006). A previous study found that women with high CCDC69 expression had longer survival times than those with low CCDC69 expression, and during cisplatin exposure, CCDC69 promoted the accumulation of p53 by activating p14ARF while inactivating MDM2 signaling to maintain p53 and p14ARF expression, suggesting that CCDC69 might be a potential therapeutic target in cancer (Cui et al., 2019). DOCK2 is a cytoplasmic protein and member of the DOCK-A subfamily of guanine exchange factors (GEFs) specific for Rac1 and Rac2 (Rac1/2) that is expressed primarily in leukocytes (Nishihara et al., 1999; Cote and Vuori, 2002). It can also regulate the functions of innate immune cells such as the migration and interferon (IFN) secretion of plasmacytoid dendritic cells, the cytotoxicity of natural killer (NK) cells, and the reactive oxygen species (ROS) production of neutrophils (Chen et al., 2018b). DOCK2 mutations have been observed in many cancers, including chronic lymphocytic leukemia (CLL), prostate cancer, and acute myeloid leukemia (AML; Hasan et al., 2018; Bjerre et al., 2019; Hu et al., 2019). In particular, DOCK2 might be a potential druggable target for AML with FLT3/internal tandem duplication (ITD) mutations (Wu et al., 2017). IKZF1 is encoded by Ikaros, which acts as a transcription factor that can control the specification and differentiation of lymphocytes (Chen et al., 2019). Overexpression of IKZF1 can activate autoimmune susceptibility through infiltrating NKG2D+ and CD8+ T cells (Chen et al., 2018a). Its prognostic value has been noted in osteosarcoma (Zhang and Yang, 2018). JCHAIN encodes the immunoglobulin J chain and links monomer units of IgA and IgM, and the upregulation of JCHAIN was likely related to tumor aggression, as studied in a cohort of patients with acute lymphoblastic leukemia (ALL) who had died (Tomar et al., 2019). NCKAP1L encodes a member of the HEM family of tissue-specific transmembrane proteins and is only expressed in hematopoietic cells. However, the biological roles of JCHAIN and NCKAP1L in human cancer have rarely been investigated.

The main limitation of this study is that only one GEO dataset was used for verification. In particular, this cohort lacked useful follow-up information, which may lead to potential bias in the data analysis. Thus, further investigations on these prognostic biomarkers and more samples with outcome data are needed to validate our findings further.

## Conclusion

In conclusion, our results confirm that ISs are associated with the survival of luminal BC patients and provide a set of novel biomarkers that may play prognostic roles in the TME of luminal BC.

## Data Availability Statement

The datasets presented in this study can be found in online repositories. The names of the repository/repositories and accession number(s) can be found in the article/Supplementary Material.

## Author Contributions

YW and GQ contributed to the conception and design of the research. MZ and FG contributed to the acquisition of data and analysis and interpretation of data. XF contributed to statistical analysis. YW and GQ contributed to drafting the manuscript. FG, YS, and XF contributed to revision of the manuscript. GQ contributed to obtaining funding. All authors contributed to the article and approved the submitted version.

### Conflict of Interest

The authors declare that the research was conducted in the absence of any commercial or financial relationships that could be construed as a potential conflict of interest.

## Footnotes

^1^http://cancergenome.nih.gov/

^2^https://www.ncbi.nlm.nih.gov/geo/

^3^https://david.ncifcrf.gov/

^4^https://string-db.org/

## Supplementary Material

Supplementary Figure 1 The expression of survival-associated biomarkers in the high‐ vs. low-IS groups in the GEO samples.

Supplementary Figure 2 The expression of survival-associated biomarkers in the high‐ vs. low-SS groups in the GEO samples.
