# Supplementary figures and images for: Identification of Tumor Microenvironment-Related Prognostic Biomarkers in Luminal Breast Cancer
Source: Front Genet. 2020 Nov 10;11:555865. doi: 10.3389/fgene.2020.555865 (PMC7735391; doi:10.3389/fgene.2020.555865)

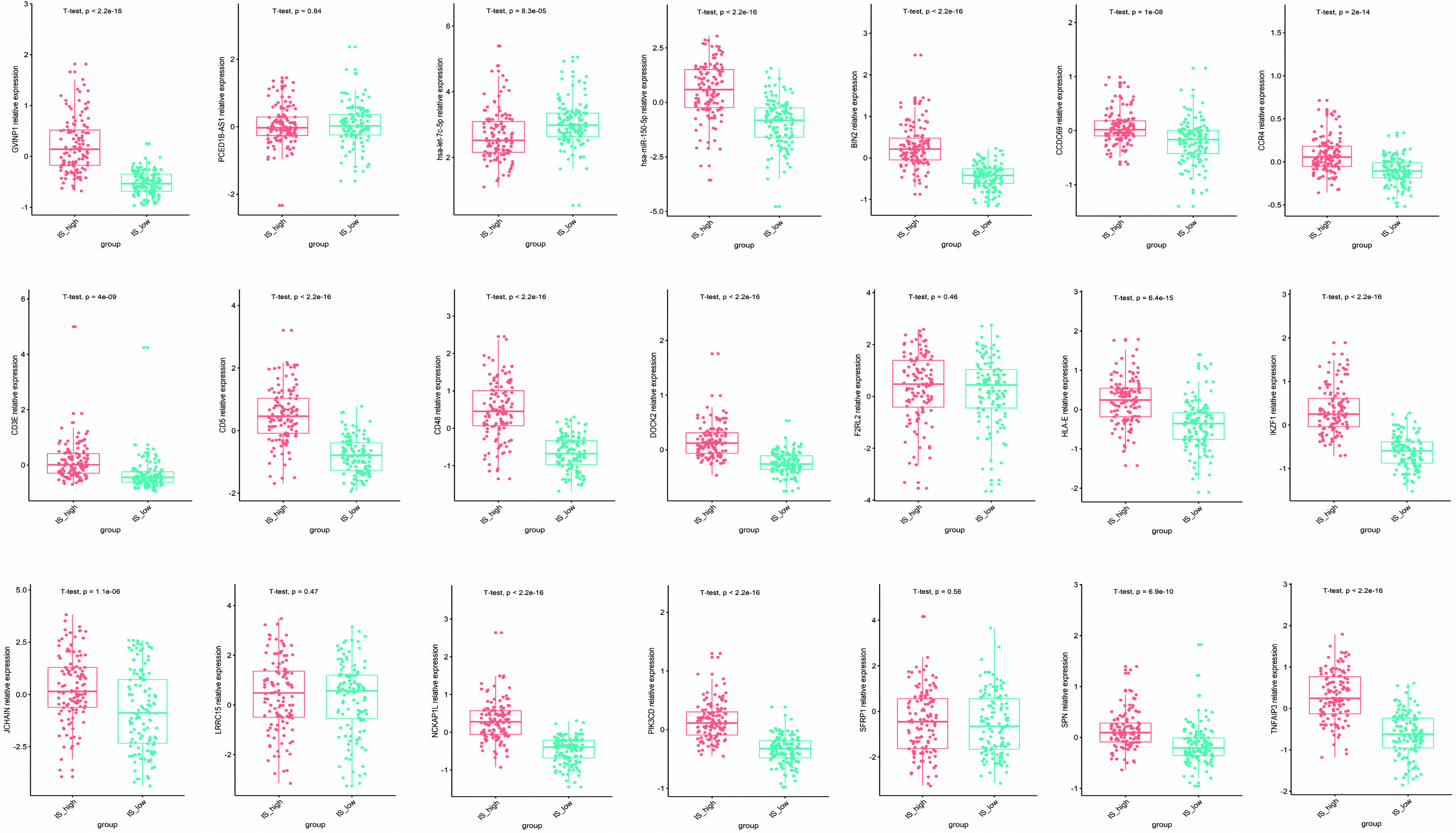

Supplement: Supplementary Figure 1 — The expression of survival-associated biomarkers in the high‐ vs. low-IS groups in the GEO samples. [file Image_1.tif]

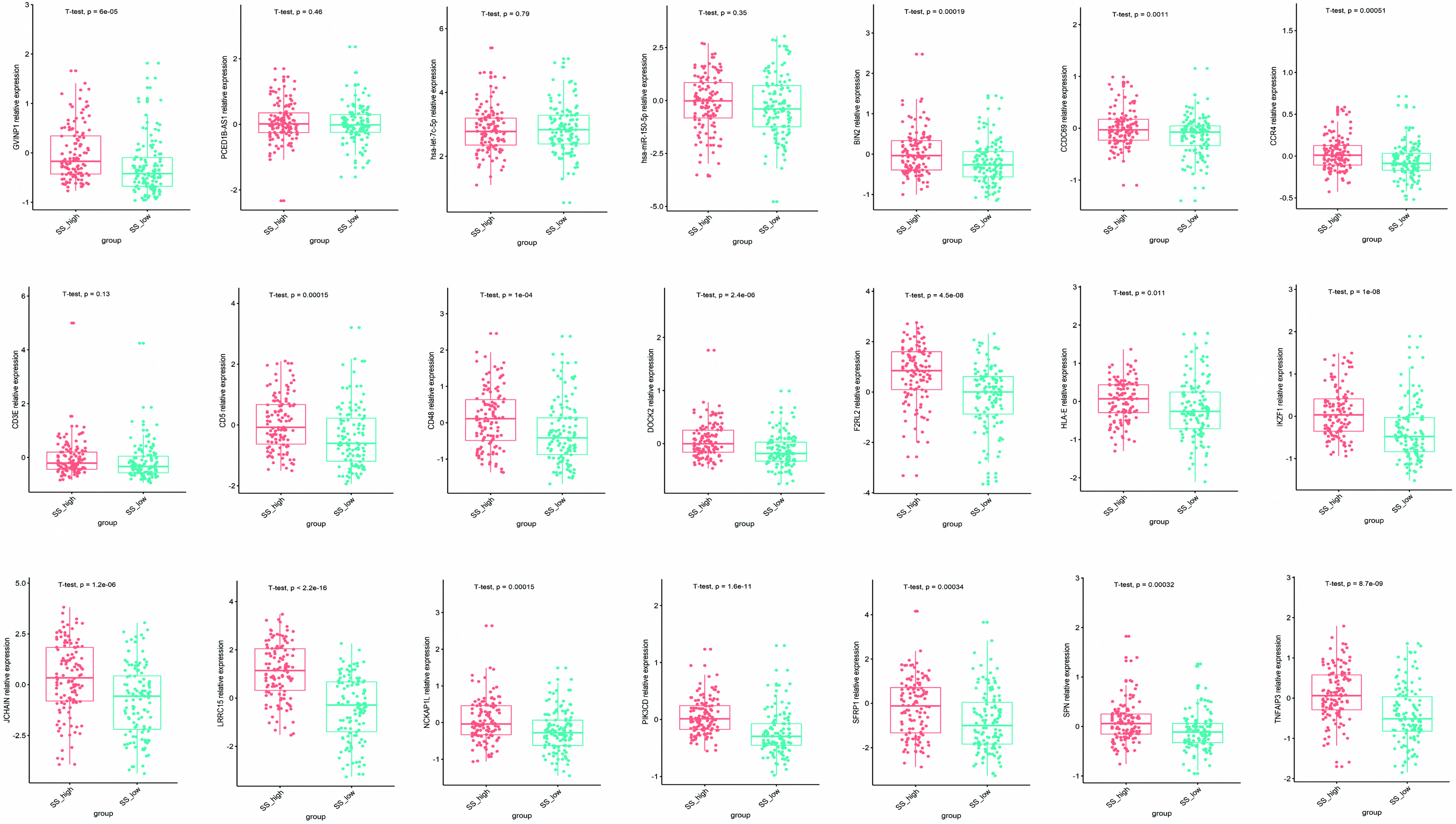

Supplement: Supplementary Figure 2 — The expression of survival-associated biomarkers in the high‐ vs. low-SS groups in the GEO samples. [file Image_2.tif]
